# Aluminum Patterned Electroplating from AlCl_3_–[EMIm]Cl Ionic Liquid towards Microsystems Application †

**DOI:** 10.3390/mi9110589

**Published:** 2018-11-12

**Authors:** Muhammad Salman Al Farisi, Silvia Hertel, Maik Wiemer, Thomas Otto

**Affiliations:** 1 Department of Robotics, Graduate School of Engineering, Tohoku University, Sendai 980-8579, Japan; 2 Fraunhofer Institute for Electronic Nano Systems (ENAS), 09126 Chemnitz, Germany; silvia.hertel@enas.fraunhofer.de (S.H.); maik.wiemer@enas.fraunhofer.de (M.W.); thomas.otto@enas.fraunhofer.de (T.O.); 3 Center for Microtechnologies (ZfM), Chemnitz University of Technology, 09126 Chemnitz, Germany

**Keywords:** aluminum, electro-chemical deposition, ionic liquid, recurrent galvanic pulse, patterned deposition, EMIC

## Abstract

Electroplating process is being used to deposit a relatively thick film of metallic materials for various microsystems applications, such as for the wafer-level bonding sealing frame and as a thermal actuator. Recently, the Al electroplating process from ionic liquid has been an attractive deposition method for anti-corrosion coatings. To extend the utilization of the film, in particular for microsystems application, a microstructure formation by patterned electroplating of Al from AlCl${}_{3}$–1-ethyl-3-methylimidazolium chloride ((EMIm)Cl) ionic liquid is investigated in this study. The influences of each deposition parameters to the electroplating process as well as the resulting surface morphology are evaluated. Electroplated Al deposits on both Au and Al seed layers are both studied. It is also found that a recurrent galvanic pulse plating process yields in a higher current efficiency. Finally, Al electroplating on a 2 µm-trenched 100 mm-wafer is also demonstrated.

## 1. Introduction

Electroplating is an essential process to deposit metallic materials for a microsystems application. For instance, various methods for integrating micro-electro mechanical systems (MEMS) and complimentary metal-oxide semiconductor (CMOS) large scale integration (LSI) utilizing metallic materials have been studied. Surface activated bonding (SAB) was used for integration as well as hermetic packaging for different devices [1]. The bonding mechanism of SAB relies on the atomic interdiffusion between different layers that have a small surface roughness and was activated using an Ar ion-beam prior to the bonding step. Electroplated Au is smoothed using either lift-off or imprint method before bonded at room temperature by SAB method [2]. Electroplated Cu is used for hermetic sealing using SAB at room temperature after chemical-mechanical polishing for surface planarization [3].

Since SAB requires a special expensive equipment, the low-temperature welding method has also been introduced as a solution for device integration and vacuum packaging. Its mechanism relies on the large-scale plastic deformation of the electroplated metallic sealing frame at the bonding interface, thus making this method insensitive to surface roughness. In this method, a device wafer with electroplated metal frames is pressed together at a high pressure with a cap wafer containing corresponding grooves. Therefore, the metallic sealing frames are wedged into the grooves and plastically deformed, inducing vacuum sealing of the enclosed cavities. Using Au, hermetic sealing at room temperature was realized [4]. Using 8 µm width Cu, hermetic sealing at 250 ${}^{\circ }$C was demonstrated [5].

Another alternative is using fly-cut planarization to compensate the topography of the electroplated metal. Wafer-level hermetic thermo-compression bonding using electroplated Au, Ag and Cu planarized by fly-cutting has been introduced [6,7,8]. These technologies have an advantage in the integration of micro-structured wafers with electrical interconnection and hermetic sealing at the same time. However, these materials are not desired in the CMOS process because they can contaminate the device. As a replacement, Al is expected due to its CMOS process compatibility.

Studies related to Al wafer bonding have been introduced [9] as a CMOS-friendly alternative to Au and Cu based bonding technologies [10,11]. However, existing studies are limited to the utilization of sputter-deposited Al film. Electroplated metallic films have provided many advantages as alternatives for wafer bonding depending on the applications as mentioned above. However, the study of Al electroplating technique for microsystems application is still limited [12].

Another example of the utilization of electroplated metal is as a thermal actuator [13]. Compared to the conventional thermal actuator made of polysilicon, metallic materials have a potential as a thermal actuator due to its higher coefficient of thermal expansion (CTE). Ni and Cu have been introduced as alternatives for the conventional polysilicon thermal actuator [14]. Both metals were deposited by electroplating to achieve a relatively thick layer. Furthermore, deposition as necessary can be realized by patterned electroplating, which also reduces the fabrication cost.

Al is also a potential replacement to the conventional polysilicon as a material for a thermal actuator. It has 10 times higher CTE compared to Si or the polysilicon [13]. However, studies regarding the patterned electroplating technique for Al is still limited.

Utilization of ionic liquid as a medium has enabled the electro chemical deposition of elements that are impossible to reduce in aqueous media, such as Al [15] and its alloys [16]. Ionic liquids are room-temperature molten salts, composed mostly of organic ions that may undergo almost unlimited structural variations [17]. After its initial developments on tungsten [18], Al films in macro-scale have been evaluated as a protective layer for structural materials such as carbon steel [19], lithium alloys [20] and magnesium alloys [21]. The anticorrosion properties of Al-coatings are based on the formation of a dense passivation layer composed by aluminium oxide that prevents further corrosive action towards the covered metal.

For such purpose, the anticorrosion ability of the Al-coatings is utilized [22], requiring the formation of a homogeneous and crack-free layer [23]. To achieve such quality, the effects of various deposition parameters have been evaluated, such as the composition of the ionic liquids [24,25,26], deposition current [27,28,29], electrolyte temperature [19], solution stirring [30] and sonication [31]. The usage of several additives has also been proposed [32] to achieve smooth and thick aluminium coatings. However, for the industrialization of the Al electroplating process, the usage of additive-free chloroaluminate ionic liquids gives a better balance between the quality of the deposit and the durability of the electroplating bath.

Patterned electroplating process allows only a required amount of a material to be deposited, not requiring an etching process to realize the desired structure. By depositing materials as required, the cost and production time can be reduced in compared to the blanket electroplating process. However, electroplating through geometrically complicated lithographic patterns presents many challenges. The difficulties are related to the electrolyte penetration and ionic transport within the defined pattern, which may result in a different behavior compared to the blanket deposition process. Furthermore, photoresist compatibility is also an issue to avoid resist degradation and unwanted dimensional change during the electroplating process [33].

On the other hand, considering CMOS process compatibility, the seed layer also has to be considered. Al is ideally chosen as a seed layer to maintain the CMOS compatibility. However, Al readily forms a native oxide under atmospheric pressure. This native oxide often blocks the electric current, not allowing material deposition on top of it. Therefore, a pre-treatment has to be performed to remove the native oxide prior to the electroplating process.

This study provides an investigation of Al patterned electroplating from AlCl${}_{3}$–[EMIm]Cl ionic liquid to form microstructures. Pattern formation is realized by a commercial negative photoresist, AZ125NXT. The influence of different deposition parameters of the patterned electroplating process on the resulting film is studied. Furthermore, reverse pulse is implemented to form an adherent film on Al seed layer. Finally, to extend the application window of the technology, the patterned electroplating process is implemented on a microstructured wafer.

## 2. Experimental Method

**Figure 1:**
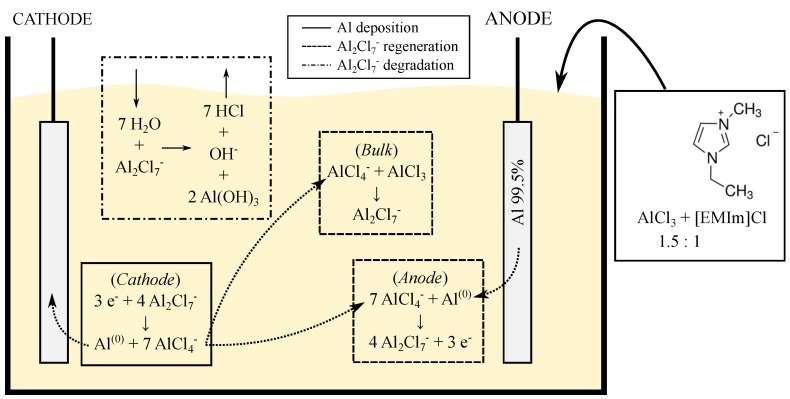
Schematics of the electrolyte used in this study and the Al electroplating process from ionic liquid.

The Al film is deposited from a commercially available AlCl${}_{3}$–[EMIm]Cl, an acidic ionic liquid with 1.5:1 concentration ratio (EP-0001, IoLiTec GmbH, Heilbronn, Germany). The structure of [EMIm]Cl ionic liquid and reactions occurring during the deposition process are as illustrated in Figure 1. The deposition of Al occurs at the cathode by a reduction process from the electroactive species Al${}_{2}$Cl${}_{7}^{-}$. The electroactive species is then regenerated by Al dissolution and reaction with AlCl${}_{4}^{-}$ at the anode. At the same time, there is also a parasitic reaction occurs between the electroactive species and the H${}_{2}$O existing in the surrounding environment. This parasitic reaction produces HCl acidic gas and accelerates the degradation of the electrolyte. Therefore, the electrolyte has to be carefully handled within an inert and dry atmosphere inside a glovebox. An experiment under ambient atmospheric environment has been reported [34]; however, a layer of decane floating on the ionic liquid is required, where the setting up has to also be done inside an inert atmosphere, which makes the process more complicated.

The deposition is performed by current-controlled electroplating using a two-electrode system (VersaSTAT 3, Princeton Applied Research, Oak Ridge, TN, USA) and a continuous magnetic bar stirring. A 99.5% Al plate is used as the anode. As the cathode, Au is initially selected as a seed layer on a Si substrate due to its stability under atmospheric pressure. In further experiments, Al is also evaluated as a seed layer on an Si substrate since Au is sometimes not accepted in process lines.

For the patterned deposition, AZ125NXT (Microchemicals, Ulm, Germany) negative photoresist is used to form 20 µm and 50 µm width patterns. The deposition process is carried out under N${}_{2}$ environment inside a commercial glovebox (LABstar, MBRAUN, Garching bei München, Germany). Around 10 µm thick film is deposited in each experiment for evaluation. No damage on the photoresist was observed even after 140 min of the deposition process.

**Figure 2:**
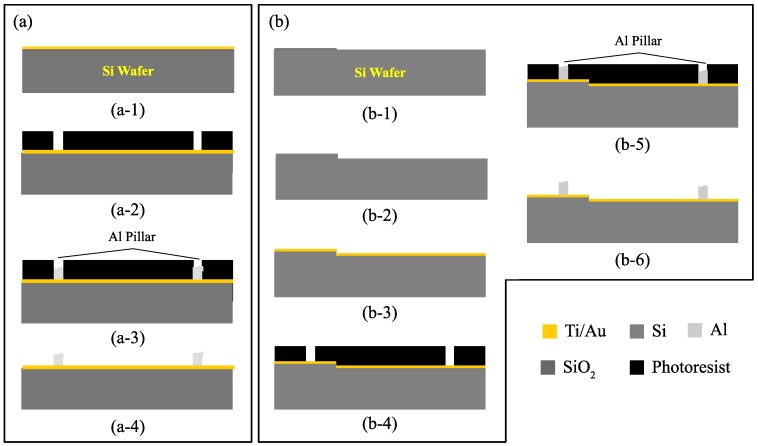
Sample fabrication process of (**a**) patterned electroplating experiment and (**b**) patterned electroplating on microstructured wafer; (**a-1**) seed layer sputter deposition on a Si wafer; (**a-2**) negative photoresist AZ125NXT patterning for electroplating; (**a-3**) Al patterned electroplating; (**a-4**) photoresist stripping; (**b-1**) thermal oxidation and SiO${}_{2}$ patterning; (**b-2**) TMAH wet etching of Si and SiO${}_{2}$ removal; (**b-3**) seed layer sputter deposition on a Si wafer; (**b-4**) negative photoresist AZ125NXT patterning for electroplating; (**b-5**) Al patterned electroplating; (**b-6**) photoresist stripping.

**Figure 3:**
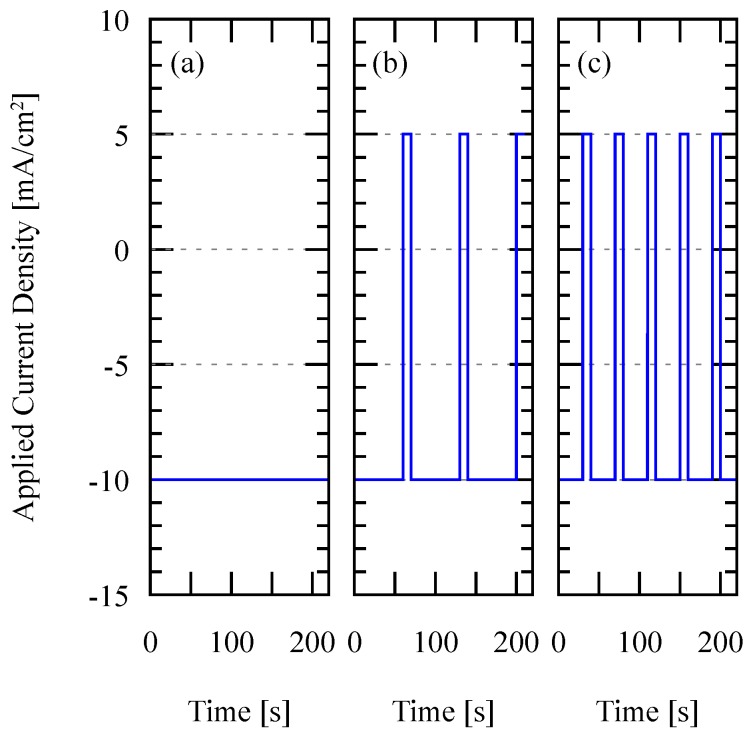
Deposition current density as a function of time in (**a**) pure DC deposition; (**b**) RGP deposition with 60 s forward and 10 s reverse current duration and (**c**) RGP deposition with 30 s forward and 10 s reverse current duration. Negative current density indicates the deposition process.

**Figure 4:**
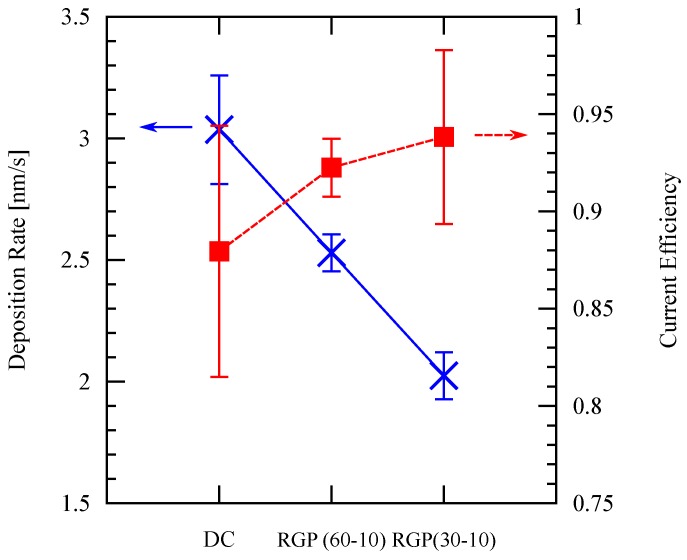
Deposition rate and current efficiency using full DC deposition and different RGP patterns shown in Figure 3. The deposition is performed at room temperature.

Fabrication steps of samples for patterned electroplating is shown in Figure 2. Figure 2a shows a patterned electroplating experiment on a standard flat wafer. The process starts by seed layer sputter deposition, either 20 nm/50 nm of Ti/Au or 1000 nm of Al, as shown in Figure 2a-1. Then, AZ125NXT negative photoresist is patterned on top of the wafer as depicted in Figure 2a-2 followed by dicing into 20 mm × 40 mm chips. The Al electroplating process is then performed at the chip level followed by photoresist stripping as illustrated in Figure 2a-3,a-4, respectively. The samples were dipped for several minutes before starting the electroplating process to ensure that the electrolyte is able to penetrate through the patterns and reach the surface of seed layer.

**Figure 5:**
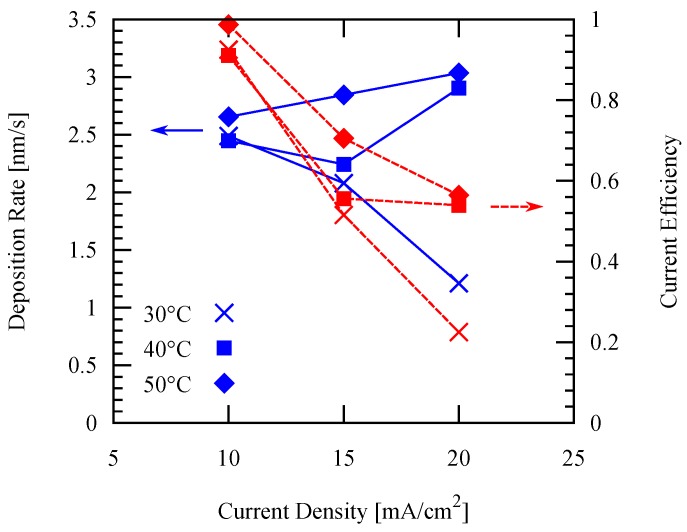
Deposition rate and current efficiency using different current densities at different electrolyte temperatures with 60 s/10 s forward/reverse current duration.

**Figure 6:**
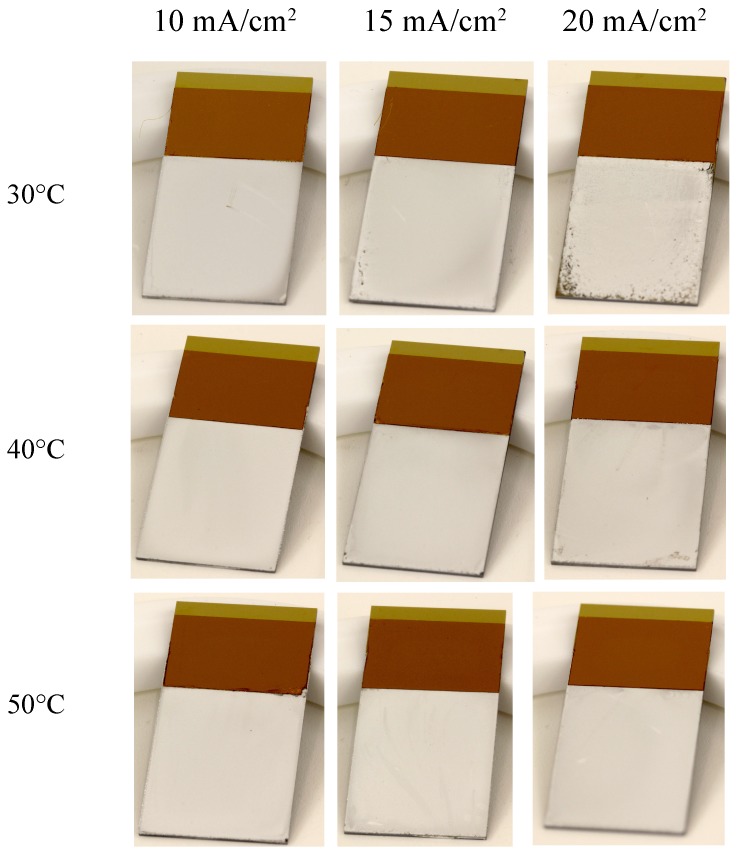
Optical photographs of the deposited bare Al under various deposition conditions.

For samples for patterned electroplating on microstructured wafer, the fabrication process is shown in Figure 2b. The process starts by 500 nm thermal oxidation of the Si wafer and patterning of the thermal SiO${}_{2}$ as shown in Figure 2b-1. Then, the Si wafer is wet etched using tetramethylammonium hydroxide (TMAH) solution to form 2 µm deep trenches and realize a microstructured wafer and the thermal SiO${}_{2}$ layer is removed. Seed layer for Al electroplating is then deposited by sputter deposition of either 20 nm/50 nm of Ti/Au or 1000 nm of Al, as shown in Figure 2b-3. Then, AZ125NXT negative photoresist is patterned on top of the wafer as depicted in Figure 2b-4, followed by Al electroplating and photoresist stripping as illustrated in Figure 2b-5,b-6, respectively.

The deposition rate and current efficiency of the bare deposits are evaluated by measuring and comparing the weight of each sample before and after deposition, i.e., gravimetric analysis. The thickness of the patterned films is evaluated by a mechanical surface profiler (AlphaStep 500, KLA-Tencor, Milpitas, CA, USA). The resulting patterned films are then characterized by scanning electron microscopy (SEM, SUPRA 60, Carl-Zeiss, Jena, Germany or SU-70, Hitachi, Japan) and their surfaces are analyzed by white light interferometry (NewView 6300, Zygo, Middlefield, CT, USA).

## 3. Results and Discussion

### 3.1. Recurrent Galvanic Pulse Electroplating

Recurrent galvanic pulse (RGP) electroplating is an electroplating process in which the forward and reverse currents are alternately applied in a relatively longer period compared to the standard pulse plating. Unlike the direct current (DC) plating, in which a constant current is used for the complete electroplating process resulting in a constant deposition of material, in RGP both deposition and the etching (reverse deposition) process is applied on the film. In this study, the etching current is set as half of the deposition current. The difference of the current density profile is visualized in Figure 3.

Here, the current efficiency η is defined as the ratio of the experimentally obtained amount of deposit to its theoretical calculation according to Faraday’s equation. It is mathematically expressed as shown in Equation (1). The measured difference of mass after and before the deposition is expressed as Δm, *F* expresses Faraday’s constant (96,485 C mol${}^{-1}$), *z* is the valency number of ions of the substance, which is 3 for Al, *I* and *t* show the current and time used for the deposition, respectively, and *M* is the molar mass of the substance, which is 26.98 gram/mol for Al:

(1) $$\eta =\frac{\Delta mFz}{ItM}.$$

The deposition rate ν is defined as the thickness of the deposit obtained for every unit of time. It is mathematically expressed as shown in Equation (2). The measured difference of mass after and before the deposition is expressed as Δm, *A* shows the deposition surface area and *t* is the total deposition duration:

(2) $$\nu =\frac{\Delta m}{At}.$$

In this study, two kinds of RGP plating conditions are applied: 60 s/10 s and 30 s/10 s forward/ reverse current duration. Both conditions are compared to the full DC electroplating while keeping the theoretical yield and other deposition parameters constant. The result is presented in Figure 4, showing that reducing the forward current duration results in a higher current efficiency but a lower deposition rate. The lower deposition rate is natural since the ratio between the deposition duration and etching duration is lower, therefore the RGP plating condition has to be compromised well to optimize both the deposition rate and the current efficiency. Based on these results, the following experiments are performed with the 60 s/10 s forward/reverse current duration. The magnitude of the reverse current density is a half the magnitude of the forward current density, or otherwise stated.

### 3.2. Effect of Temperature and Current Density

Bare substrates are used to evaluate the effect of the temperature and current density on the resulting deposit. Many reports exist on the effect of temperature and current density to the resulting deposit, and Chang et al. [21] investigated the room temperature deposition process. With a voltage controlled deposition, it was found in the same report that a high deposition potential will result in a high deposition rate, which would bring out a loose structure and small cracks within the Al layer.

Electrodeposits obtained using DC plating between 10 and 70 mA/cm${}^{2}$ were found to be quite dense and adhere well to the Al substrates [27]. However, dendrite growth with relatively poor adherence was obtained at over 100 mA/cm${}^{2}$ current densities. The current efficiency increases from 85% to nearly 100% as the current density increases from 10 to 40 mA/cm${}^{2}$. When the current density was raised further, the current efficiency decreases to a constant value of approximately 92% between 50–100 mA/cm${}^{2}$. During deposition at higher current densities, the ionic liquid also became less chemically stable.

According to Berretti et al. [31], a higher electrolyte temperature resulted in a higher conductivity of the electrolyte, and thus a higher deposition rate. However, the ionic liquid is decomposed at a temperature higher than 60 ${}^{\circ }$C. Considering these prior studies, in this study, the temperature is varied between 30–50 ${}^{\circ }$C with 10 ${}^{\circ }$C step and the current density is varied between 10–20 mA/cm${}^{2}$ with a 5 mA/cm${}^{2}$ step.

Deposition rate and current efficiency as a function of the current density are shown in Figure 5. Deposition rate is generally increased by increasing the current density. However, in this study, poor current efficiency and film uniformity were obtained at higher current densities. This can be attributed to the side reactions due to a high potential at the applied current densities. Especially because blanket substrates are used, side reactions also occur at the corners. This is reflected from the optical photograph of each sample shown in Figure 6.

**Figure 7:**
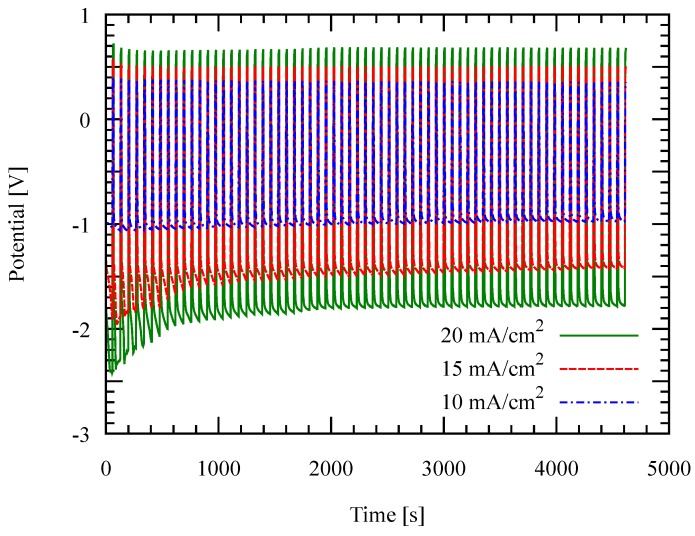
Deposition potential as a function of time during the deposition at 30 ${}^{\circ }$C with different current densities. Negative potential shows the deposition potential.

**Figure 8:**
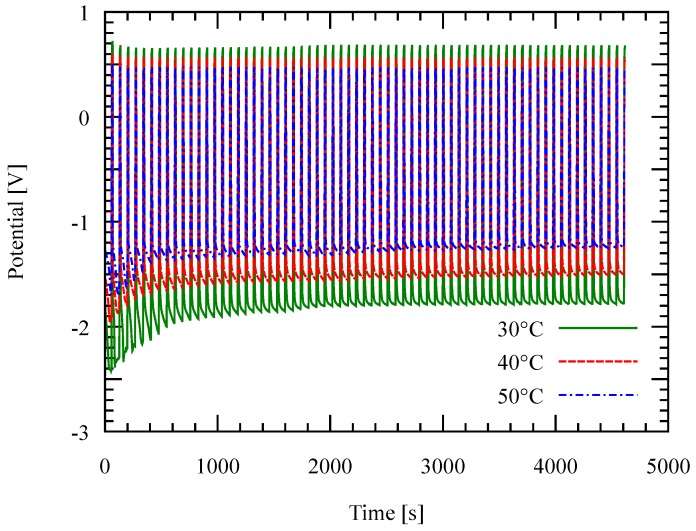
Deposition potential as a function of time during the deposition at different temperatures with a fixed current density of 20 mA/cm${}^{2}$. Negative potential shows the deposition potential.

Both the deposition rate and the current efficiency decrease with the current density at 30 ${}^{\circ }$C. This can be attributed to the high resistance of the electrolyte which enhances side reactions. Consequently, high deposition potential was observed at higher current density as shown in Figure 7. Conductivity of ionic liquid generally increases with the temperature. This case is confirmed in Figure 8; the deposition potential decreases as the temperature is increased.

**Figure 9:**
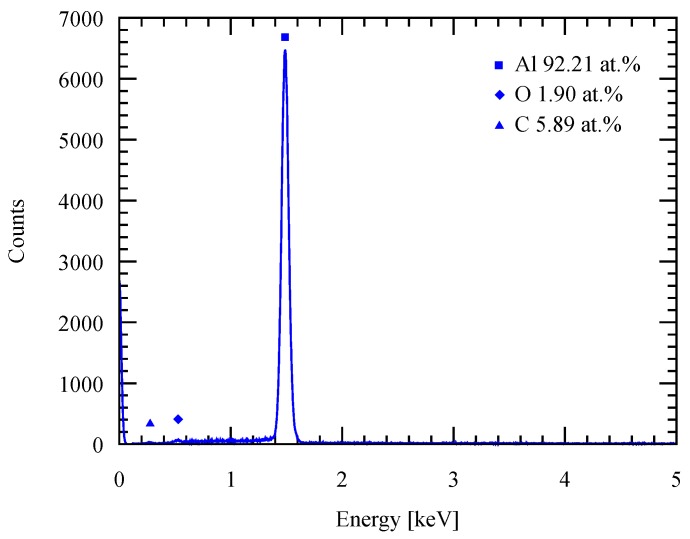
EDS spectrum of the deposited film with 20 mA/cm${}^{2}$ current density at 50 ${}^{\circ }$C.

To evaluate the purity of the deposit, an energy-dispersive X-ray spectroscopy (EDS) was performed on the deposits as shown in Figure 9. According to the EDS result, an Al deposit with high-purity is obtained from the electroplating process. Oxygen naturally exists due to the high reactivity of Al with oxygen, which forms native oxide on the surface as soon as it is exposed to the atmospheric environment. No trace of Cl shows that there is no ionic liquid residue that remains on the deposit, while C atoms can be attributed to the measurement environmental effects, i.e., the SEM stage.

### 3.3. Patterned Electroplating

**Figure 10:**
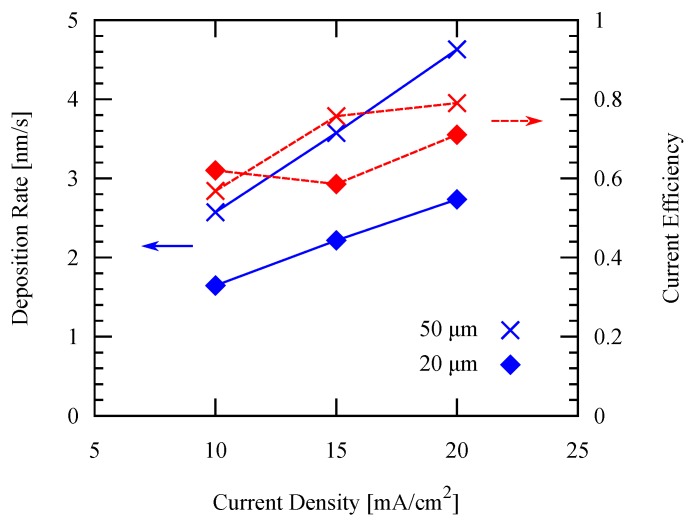
Deposition rate and current efficiency using different current densities applied with different pattern widths.

**Figure 11:**
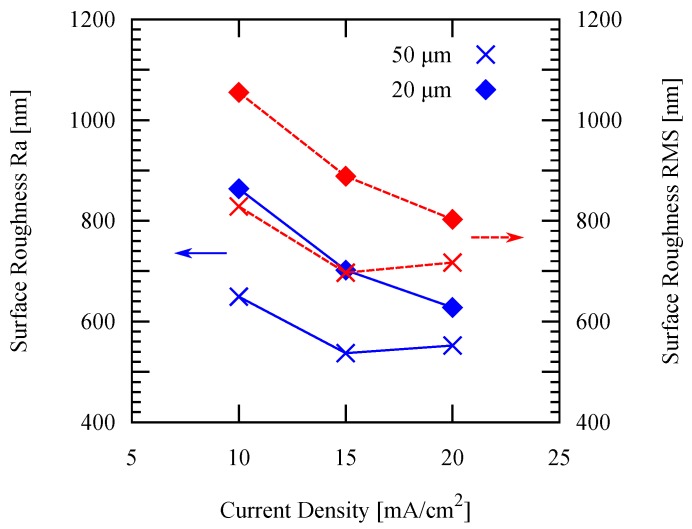
Surface roughness of the resulting deposits using different current densities applied with different pattern widths.

For the patterned electroplating experiments, the temperature is fixed at 50 ${}^{\circ }$C, while two different pattern widths are applied: 50 µm and 20 µm. It is shown in Figure 10 that both the deposition rate and the current efficiency increase by increasing the current density. Furthermore, both the arithmetic average surface roughness Ra and root mean squared (RMS) roughness measured by white light interferometry decreases as the current density increases as shown in Figure 11. These values are in agreement with those values of previous studies mentioned in the previous section, which showed similar trends.

**Figure 12:**
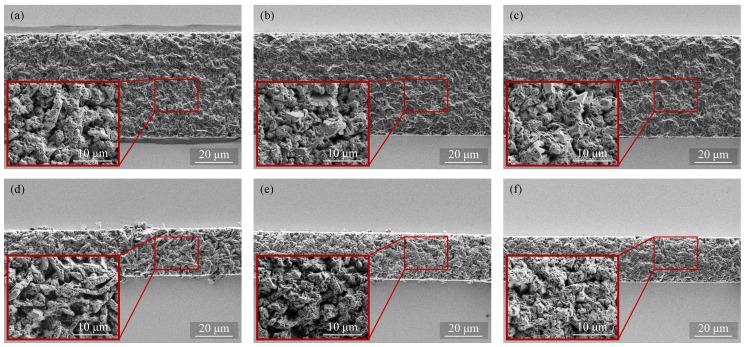
SEM images of the pattern-deposited films with 50 µm frame width obtained at (**a**) 10; (**b**) 15; (**c**) 20 mA/cm${}^{2}$ and with 20 µm frame width obtained at (**d**) 10; (**e**) 15; and (**f**) 20 mA/cm${}^{2}$.

Figure 12 shows SEM images of the deposited layers obtained with each frame width and current density. As shown in Figure 12a,d, large dendritic granules can be observed at the surfaces of the deposits obtained at lower current density. Smaller grain is obtained by increasing the applied current density. This observation of the grain size explains the decreasing surface roughness with the increase of the current density.

**Figure 13:**
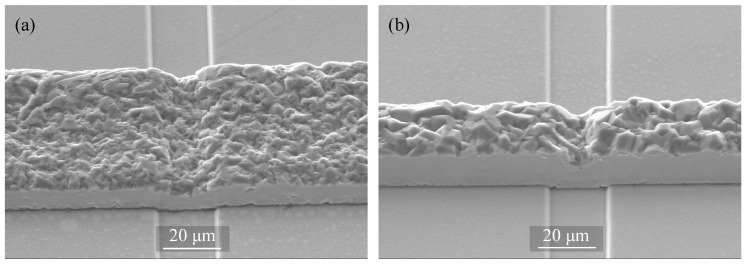
SEM images of the pattern-deposited films on a 2 µm–depth trenched substrate with (**a**) 50 µm and (**b**) 20 µm frame width obtained at 20 mA/cm${}^{2}$.

Another deposition experiment is demonstrated on micro-structured substrates, with 2 µm-depth trenches, aiming for wider application window in the microsystem world. To demonstrate the ability of the electroplating process for batch fabrication, the experiment is performed at the wafer-level on a 100 mm-diameter wafer, which is one of the standard wafer sizes for micro-device fabrication. The SEM images of the resulting deposits are depicted in Figure 13.

### 3.4. Al Seed Layer

**Figure 14:**
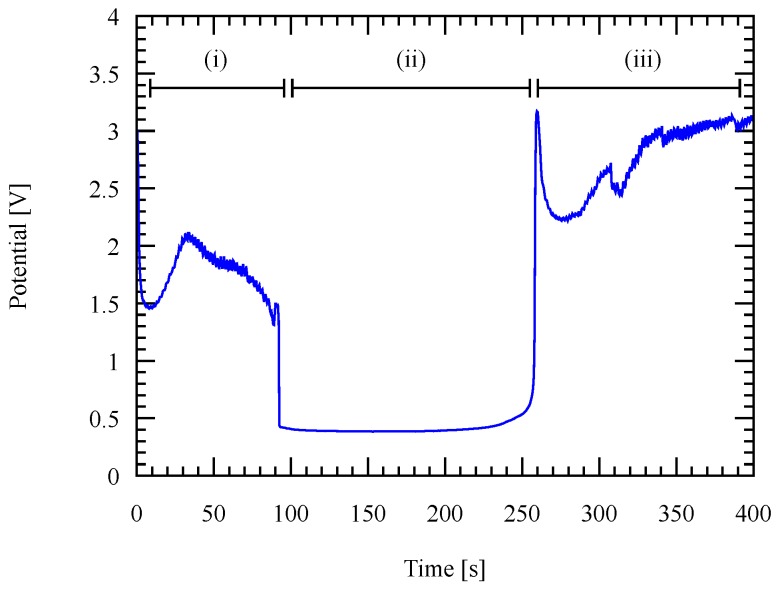
Reverse deposition potential as a function of time during the reverse current treatment to remove the native oxide layer on Al seed layer with a fixed current density of 10 mA/cm${}^{2}$.

The main difference between Al and Au seed layers is that Al is easily oxidized under atmospheric exposure. To conduct a successful electroplating process, a pure metalic surface is required to conduct the deposition current properly. Therefore, a treatment to remove the native oxide has to be performed prior to the deposition process. In this study, substrates with Al seed layer are treated with a reverse current before the deposition process starts. A full reverse current profile of 1 µm thick Al seed layer is as shown in Figure 14.

Region (i) in Figure 14 corresponds to the removal process of the native oxide at the surface of the Al seed layer. Region (ii) corresponds to the pure Al surface exposure to the electrolyte and region (iii) corresponds to the depleted Al seed layer, leaving the Si substrate exposed to the electrolytes. In this experiment, all processes are done with a reverse current to completely remove the native oxide layer as in region (i) with an additional 30 s in region (ii) to have a more certainty.

**Figure 15:**
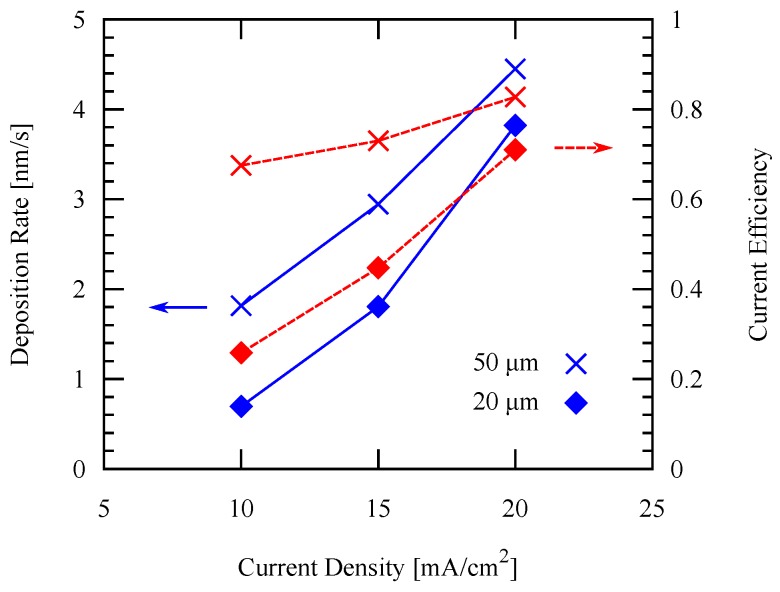
Deposition rate and current efficiency using different current densities applied with different pattern widths on an Al seed layer.

**Figure 16:**
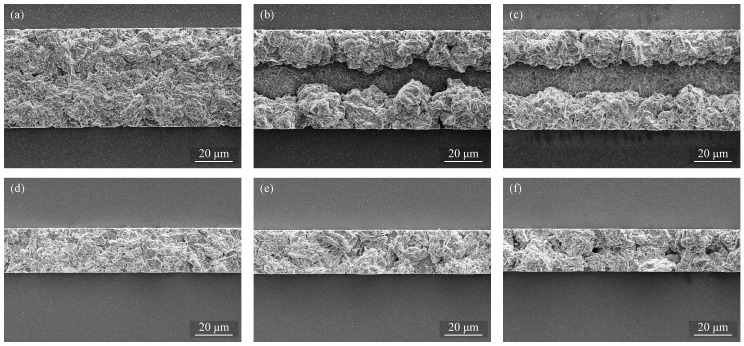
SEM images of the pattern-deposited films on an Al seed layer with 50 µm frame width obtained at (**a**) 10; (**b**) 15; (**c**) 20 mA/cm${}^{2}$ and with 20 µm frame width obtained at (**d**) 10; (**e**) 15; and (**f**) 20 mA/cm${}^{2}$.

The effect of current density to the deposition rate and current efficiency is evaluated as shown in Figure 15. This result agrees with the previous pattern plating result using Au seed layer in the previous section. SEM images of the deposited layers obtained with each frame width and current density are shown in Figure 16. It can be seen from the SEM images that the deposition is not uniform along the whole pattern. This could be caused by deposition rate non-uniformity, the rate on the sides is higher than the center, resulting in such surface profile.

## 4. Conclusions

A microstructure formation by a patterned electroplating process of Al from AlCl${}_{3}$–[EMIm]Cl ionic liquid has been demonstrated and evaluated for various deposition parameters. Due to its excellent mechanical, thermal and electrical properties, Al is one of the prospective candidates for a material for microelectronics and microsystems application.

RGP deposition resulted in a higher current efficiency than the normal DC deposition, however, with a lower deposition rate. The electroplating process at a higher temperature was also found to give a better stability in the process due to a reduction in side reactions. However, there is a limit to the temperature as the ionic liquid is decomposed at a high temperature.

In patterned electroplating, higher current density yields both higher current density and current efficiency. Native oxide removal on the Al seed layer was also demonstrated by reverse current deposition and electroplating on the Al seed layer was demonstrated. This study has a potential to broaden the usage of the Al-coating for microelectronics and microsystems applications.
